# Nanomaterials in Smart Energy-Efficient Coatings

**DOI:** 10.3390/nano14221820

**Published:** 2024-11-13

**Authors:** Xun Cao

**Affiliations:** 1 State Key Laboratory of High Performance Ceramics and Superfine Microstructure, Shanghai Institute of Ceramics, Chinese Academy of Sciences, Shanghai 200050, China; cxun@mail.sic.ac.cn; 2 Center of Materials Science and Optoelectronics Engineering, University of Chinese Academy of Sciences, Beijing 100049, China

## 1. Introduction

Temperature is a key manifestation of energy, with about 51% of global energy consumption occurring in the form of heat annually [1]. However, the current reliance on conventional fossil fuels for heating and cooling systems contributes significantly to carbon dioxide emissions, exacerbating environmental challenges. The urgent need to transition from fossil fuel dependence to renewable energy sources has led to increased interest in solar energy as a sustainable alternative [2,3,4,5]. Traditional temperature control technologies, particularly those based on radiative mechanisms, are often limited by their fixed nature and singular functionality, making them unsuitable for dynamic natural environments and diverse application scenarios [6,7]. Consequently, the development of smart energy-efficient coatings has become both necessary and timely. Smart regulation is initially applied to temperature control through electrochromism and photochromism, and dynamic radiation modulation is currently the most promising technology due to its potential for widespread application. Smart coatings, when applied to building components such as doors and windows, can regulate energy exchange between indoor and outdoor environments by controlling the transmittance, reflectivity, or emissivity of light [1,8]. Over the past decades, significant progress in nanotechnology has greatly improved the performance of dynamic color-changing materials, enhancing their practical application in energy-efficient systems.

Smart energy-efficient coatings that utilize electrochromic, thermochromic, gasochromic, and photochromic properties have been widely studied. Among them, vanadium dioxide (VO_2_) is a typical representative of thermochromic materials due to its phase transition temperature (T_C_ ≈ 68 °C). Which is close to room temperature [9,10,11]. A significant advantage of VO_2_ films is that they can modulate infrared thermal radiation through intrinsic temperature responsiveness, requiring no external energy input. However, VO_2_ is severely limited in its application because high-quality, high-performance films cannot be easily synthesized by straightforward methods. Meanwhile, the high temperatures required for the synthesis of VO_2_ films limit their application on flexible substrates. In spacecraft, thermochromic coatings are employed to radiate heat into space, and current research focuses on optimizing the balance between radiation emissivity and solar absorptivity [12]. Therefore, the development of thermochromic thin films with adaptive switching emission capabilities holds considerable significance for spacecraft technology. Electrochromic materials, despite their potential, face challenges such as the complexity of multi-layer stacking, which complicates large-area applications. Additionally, introducing mixed electrolytes can lead to the migration of ion storage materials to the surface of the electrochromic layer, thus degrading optical storage performance and resulting in leakage current. A viable solution involves integrating electron shielding and ionic conduction structures to ensure efficient electrochromic reactions. Inorganic photochromic materials have gained increasing research interest due to their high thermal stability, long cycle life, and excellent chemical resistance, making them promising candidates for durable and resilient smart coating applications.

## 2. An Overview of Published Articles

Wang et al. (Contribution 1) deposited a high-quality VO_2_ film using magnetron sputtering followed by vacuum annealing at 425 °C. This study presents a thermally switchable metamaterial absorber (TSMA) based on the phase-change material of VO_2_. The change in square resistance of the VO_2_ film during phase transition is approximately 3.65 orders of magnitude. The absorption frequency of TSMA was 7.3 GHz in the insulating state, shifting to 6.8 GHz in the metallic state. Both simulation and experimental results demonstrate that this study provides valuable insights for the application of VO_2_ switchable metasurfaces in the microwave band.

Hao et al. (Contribution 2) developed a simple and efficient hydrothermal synthesis method to prepare monoclinic VO_2_ nanorods with high crystallinity. V_2_O_5_ and H_2_C_2_O_4_ were used as raw materials to prepare the precursor solution, which was reacted at 280 °C for 48 h in an autoclave to obtain the VO_2_ nanorods, and thin films were prepared using screen-printing or spin-coating techniques on the surface of fabrics or glass. The emissivity of the prepared flexible films can reach 22%, and the prepared rigid films can maintain a visible light transmittance of over 50%, coupled with an infrared transmittance modulation exceeding 20% at 1.5 µm. These results demonstrate that the films prepared using this method can spontaneously adjust the emissivity in response to environmental changes, providing a new solution for adaptive infrared stealth applications.

Ali et al. (Contribution 3) reported the solar absorptance properties of a VO_2_-based smart radiator device (SRD). By combining optical simulations, they improved the SRD structure by adding alternating top-stacked layers of materials with high and low refractive indices, specifically the a-Si/SiO_2_ layer, to the VO_2_/SiO_2_/Au SRD structure. The modified structure reduced the solar absorptivity by 35% from 0.43 to 0.28 while achieving an emissivity modulation of up to 0.46. The VO_2_-based SRDs exhibit a promising solution for energy-saving thermal control, particularly for nanosatellites in the aerospace sector.

Song et al. (Contribution 4) achieved a high-performance electronchromic windows (ECW) based on a “hybrid electrolyte + electron barrier layer” system, utilizing a perfluorosulfonic acid (PFSA)-coated Prussian blue (PB) film combined with a Ferricyanide–Ferrocyanide-containing hybrid electrolyte. The system offered several advantages, including a minimized device voltage due to the similar redox potentials for coloring at 0.4 V and bleaching at 0.6 V, effective control over the side reaction for oxygen and hydrogen evolution, and low cost by using accessible raw materials. Additionally, the system exhibited high transparency in both the visible and near-infrared regions, ensuring excellent compatibility with electrochromic electrodes. A cyclic stability of the PFSA/PB-ECW demonstrated significant improvements in optical memory and modulation amplitude, with 76.9% of the transmittance modulation at 633 nm retained after 300 cycles. This straightforward design provides an effective way for the wide application of electrochromic devices.

Li et al. (Contribution 5) prepared inorganic photochromic films of Gd_1−z_Y_z_O_x_H_y_ (z = 1, 0.7, 0.5, 0.4, 0.35, 0.25, 0.15, 0) through a one-step direct-current magnetron co-sputtering process. By alloying rare earth cations, they engineered the lattice constants to modulate the photochromic properties and stability of the Gd_1−z_Y_z_O_x_H_y_ films. As the lattice constant increased, the photochromic properties became better, with the best photochromic performance with the lattice constant of 5.51 Å, resulting in an optical contrast of 37%, which was a 37.1% improvement compared to GdO_x_H_y_ film. This work provides insights into the development of innovative photochromic materials, offering a promising direction for future applications of rare earth oxyhydride films.

## 3. Conclusions

In conclusion, the Special Issue emphasizes the latest theoretical developments and practical applications of smart energy-efficient coatings, aiming to engage both academic and industrial researchers in fostering the development of these innovative materials for energy-efficient applications. The recent emergence of nanostructures, facilitated by advancements in nanotechnology, offers significant opportunities to enhance the performance of smart energy-efficient coatings. Furthermore, nanostructure designs have been developed to modulate properties in the infrared region, thereby expanding their applicability to spacecraft. The introduction of composite nanomaterials, incorporating both organic and inorganic components, has also demonstrated flexible performance, which is expected to promote the broader adoption of smart energy-efficient coatings.

Additionally, it is important to recognize that future practical applications will require more intelligent management systems capable of delivering both heating and cooling effects. These systems should be able to intelligently switch between modes with zero energy consumption, responding dynamically to environmental changes. The realization of truly zero-energy dual-mode devices holds significant potential for global thermal management and energy conservation, providing a promising pathway toward achieving net-zero carbon emissions goals in the future.
